# Poly[(μ_4_-5-bromo­pyridine-3-sulfonato)­silver(I)]

**DOI:** 10.1107/S1600536811055206

**Published:** 2012-01-07

**Authors:** Ying-Bing Lu, Fang-Mei Jian

**Affiliations:** a College of Chemistry and Chemical Engineering, Gannan Normal University, Ganzhou 341000, People’s Republic of China

## Abstract

The silver(I) complex, [Ag(C_5_H_3_BrNO_3_S)]_*n*_, was obtained by reaction of AgNO_3_ and 5-bromopyridine-3-sulfonic acid. The Ag^I^ ion is coordinated by an O_3_N donor set in a slightly distorted tetra­hedral geometry. The Ag^I^ ions are linked by μ_4_-5-bromo­pyridine-3-sulfonate ligands, forming a layer parallel to (100). The layers are further connected *via* C—H⋯Br hydrogen-bonding inter­actions into a three-dimensional supra­molecular network. The Ag⋯Ag separation is 3.0159 (6) Å, indicating the presence of argentophilic inter­actions.

## Related literature

For background information on pyridine­sulfonato ligands, see: Chandler *et al.* (2002 ▶); Makinen *et al.* (2001 ▶); May & Shimizu (2005 ▶). For similar C—H⋯Br hydrogen bonding, see: Lu *et al.* (2011 ▶).
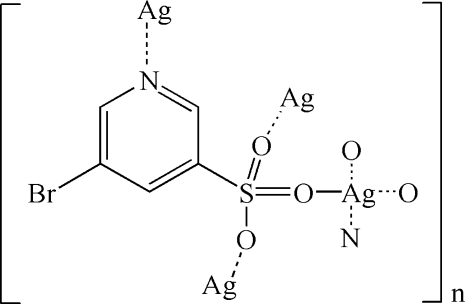


## Experimental

### 

#### Crystal data

[Ag(C_5_H_3_BrNO_3_S)]
*M* *_r_* = 344.92
Monoclinic, 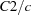

*a* = 20.103 (3) Å
*b* = 5.0634 (9) Å
*c* = 16.036 (3) Å
β = 110.142 (2)°
*V* = 1532.5 (5) Å^3^
*Z* = 8
Mo *K*α radiation
μ = 8.08 mm^−1^
*T* = 296 K
0.20 × 0.18 × 0.16 mm

#### Data collection

Bruker SMART CCD area-detector diffractometer
Absorption correction: multi-scan (*SADABS*; Sheldrick, 2008*a* ▶) *T*_min_ = 0.512, *T*_max_ = 0.746
4188 measured reflections
1310 independent reflections
1204 reflections with *I* > 2σ(*I*)
*R*_int_ = 0.022

#### Refinement

*R*[*F* ^2^ > 2σ(*F* ^2^)] = 0.038
*wR*(*F* ^2^) = 0.152
*S* = 1.01
1310 reflections
109 parameters
2 restraints
H-atom parameters constrained
Δρ_max_ = 0.88 e Å^−3^
Δρ_min_ = −1.72 e Å^−3^

### 

Data collection: *SMART* (Bruker, 2001 ▶); cell refinement: *SAINT* (Bruker, 2001 ▶); data reduction: *SAINT*; program(s) used to solve structure: *SHELXS97* (Sheldrick, 2008*b* ▶); program(s) used to refine structure: *SHELXL97* (Sheldrick, 2008*b* ▶); molecular graphics: *SHELXTL* (Sheldrick, 2008*b* ▶); software used to prepare material for publication: *SHELXTL*.

## Supplementary Material

Crystal structure: contains datablock(s) I, global. DOI: 10.1107/S1600536811055206/zj2045sup1.cif

Structure factors: contains datablock(s) I. DOI: 10.1107/S1600536811055206/zj2045Isup2.hkl

Additional supplementary materials:  crystallographic information; 3D view; checkCIF report

## Figures and Tables

**Table 1:** Hydrogen-bond geometry (Å, °)

*D*—H⋯*A*	*D*—H	H⋯*A*	*D*⋯*A*	*D*—H⋯*A*
C3—H3*A*⋯Br1^i^	0.93	2.92	3.832 (3)	168

Symmetry code: (i) 
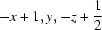
.

**Fig. 1.**
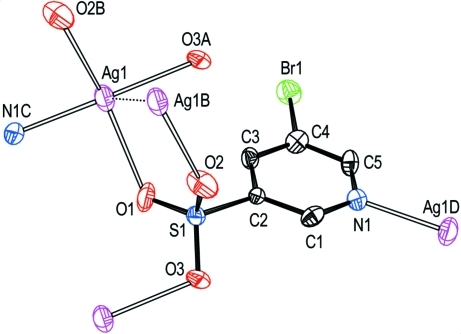
ORTEP drawing of 1 with 50% thermal ellipsoids with hydrogen atoms being omitted for clarity. (Symmetry codes: A: x, 1 + y, z; B: 1/2 - x, 3/2 - y, - z; C: x, 1 - y, -1/2 + z; D: x, 1 - y, 1/2 + z;).

**Fig. 2.**
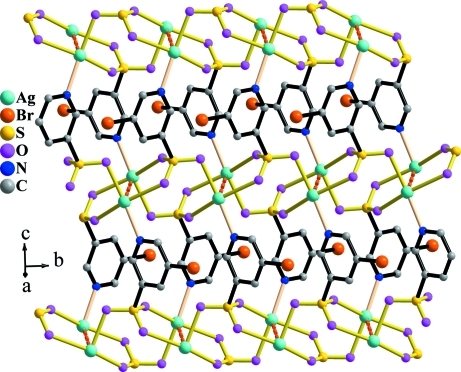
View of two-dimensional layer of 1 along the a axis. The yellow–green bonds represent the 1-D chain originating from Ag and SO3 groups of 5-Bromopyridine-3-sulfonato ligands. The silver-silver interactions are represented as orange dashed lines (H atoms are omitted for clarity).

**Fig. 3.**
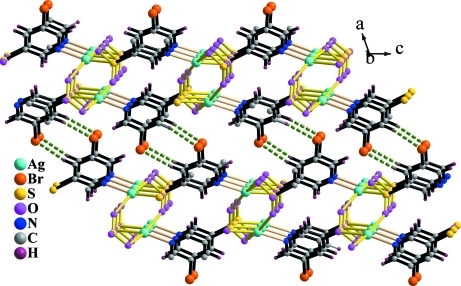
Three-dimensional supramolecular network of 1 showing C3—H3···Br hydrogen-bonding interactions (green dashed lines).

## supplementary crystallographic information

### Comment

As bridging ligands, sulfonate ligands and their derivatives have drawn much attention owing to their diverse coordination modes, forming numerous coordination complexes. In this paper, we report the new title compound 1, which displays a two-dimensional layer structure.

X-ray diffraction analyses reveal that the title compound crystallizes in the *C2/c* group space. In the asymmetrical unit of 1 (Fig. 1), there is one crystallographically independent Ag^+^ ion and one 5-Bromopyridine-3-sulfonato ligand. The Ag1 atom is in a distorted tetrahedral coordination environment and coordinated by one O1 atom, one O2 atom, one O3 atom and N1 atom from four different 5-Bromopyridine-3-sulfonato ligands. As shown in Figure 2, the Ag1 ions are linked by three oxygen atoms from sulfonate groups to form 1-D chain. Interestingly, the Ag···Ag separation in the [Ag1]2 dimers is 3.0159 (6) Å, which is much shorter than the sum of van der Waals radii for silver (3.4 Å), suggesting significant silver-silver interactions. These chains are further connected through N1 atoms from µ_4_-5-Bromopyridine-3-sulfonato ligands to generate a two-dimensional layer. The layers are connected *via* C3—H3A···Br1 hydrogen bonding interactions (Lu *et al.*, 2011) into a three-dimensional supramolecular architecture (Fig. 3 and Table 1).

### Experimental

AgNO_3_ (85 mg, 0.5 mmol) and bromopyridinesulfonato ligands (103 mg, 0.5 mmol) were dissolved in 20 ml water, stirring for 2 h. The resulting solution was filtrated and allowed to evaporate slowly at room temperature. Colorless block crystals appeared after 1 week. Yield based on Ag: 15%.

### Refinement

H atoms were placed in calculated positions with C—H = 0.93 Å (aromatic), and refined in riding mode with *U*_iso_(H) = 1.2*U*_eq_(C). The abnormal reflections (-7 1 2), (-4 2 3), (8 0 0), (1 1 6) (-4 0 2), (-2 06) and (-5 1 3) have been omitted during the refinement. The "delu 0.005 C1 N1 Ag1 O1" has been employed during the refinement to modify the small difference of anisotropic displacement parameters along chemical bonds.

### Crystal data

**Table:**

[Ag(C_5_H_3_BrNO_3_S)]	*F*(000) = 1296
*M**_r_* = 344.92	*D*_x_ = 2.990 Mg m^−^^3^
Monoclinic, *C*2/*c*	Melting point: not measured K
*a* = 20.103 (3) Å	Mo *K*α radiation, λ = 0.71073 Å
*b* = 5.0634 (9) Å	θ = 2.2–25°
*c* = 16.036 (3) Å	µ = 8.07 mm^−^^1^
β = 110.142 (2)°	*T* = 296 K
*V* = 1532.5 (5) Å^3^	Blcok, colorless
*Z* = 8	0.20 × 0.18 × 0.16 mm

### Data collection

**Table:**

Bruker SMART CCD area-detector diffractometer	1310 independent reflections
Radiation source: fine-focus sealed tube	1204 reflections with *I* > 2σ(*I*)
graphite	*R*_int_ = 0.022
phi and ω scans	θ_max_ = 25.0°, θ_min_ = 2.2°
Absorption correction: multi-scan (*SADABS*; Sheldrick, 2008*a*)	*h* = −23→23
*T*_min_ = 0.512, *T*_max_ = 0.746	*k* = −6→6
4188 measured reflections	*l* = −19→19

### Refinement

**Table:**

Refinement on *F*^2^	Primary atom site location: structure-invariant direct methods
Least-squares matrix: full	Secondary atom site location: difference Fourier map
*R*[*F*^2^ > 2σ(*F*^2^)] = 0.038	Hydrogen site location: inferred from neighbouring sites
*wR*(*F*^2^) = 0.152	H-atom parameters constrained
*S* = 1.01	*w* = 1/[σ^2^(*F*_o_^2^) + (0.132*P*)^2^] where *P* = (*F*_o_^2^ + 2*F*_c_^2^)/3
1310 reflections	(Δ/σ)_max_ = 0.009
109 parameters	Δρ_max_ = 0.88 e Å^−^^3^
2 restraints	Δρ_min_ = −1.72 e Å^−^^3^
0 constraints	

### Special details

**Table:**

Geometry. All e.s.d.'s (except the e.s.d. in the dihedral angle between two l.s. planes) are estimated using the full covariance matrix. The cell e.s.d.'s are taken into account individually in the estimation of e.s.d.'s in distances, angles and torsion angles; correlations between e.s.d.'s in cell parameters are only used when they are defined by crystal symmetry. An approximate (isotropic) treatment of cell e.s.d.'s is used for estimating e.s.d.'s involving l.s. planes.
Refinement. Refinement of *F*^2^ against ALL reflections. The weighted *R*-factor *wR* and goodness of fit *S* are based on *F*^2^, conventional *R*-factors *R* are based on *F*, with *F* set to zero for negative *F*^2^. The threshold expression of *F*^2^ > σ(*F*^2^) is used only for calculating *R*-factors(gt) *etc*. and is not relevant to the choice of reflections for refinement. *R*-factors based on *F*^2^ are statistically about twice as large as those based on *F*, and *R*- factors based on ALL data will be even larger.

### Fractional atomic coordinates and isotropic or equivalent isotropic displacement parameters (Å^2^)

**Table:**

	*x*	*y*	*z*	*U*_iso_*/*U*_eq_	
Ag1	0.325354 (14)	0.79247 (6)	0.004298 (16)	0.03990 (8)	
Br1	0.505370 (16)	0.92599 (7)	0.38247 (2)	0.03684 (10)	
S1	0.31936 (4)	0.26125 (15)	0.13294 (5)	0.0237 (2)	
N1	0.36109 (14)	0.3610 (6)	0.39356 (17)	0.0280 (7)	
O1	0.36615 (13)	0.3588 (6)	0.08721 (15)	0.0417 (6)	
O2	0.25004 (15)	0.3786 (6)	0.10181 (17)	0.0468 (8)	
O3	0.31813 (13)	−0.0272 (5)	0.13609 (16)	0.0365 (7)	
C1	0.33643 (16)	0.2719 (7)	0.3129 (2)	0.0252 (8)	
H1A	0.3026	0.1385	0.2993	0.030*	
C2	0.35925 (15)	0.3708 (6)	0.24497 (18)	0.0201 (7)	
C3	0.40983 (15)	0.5648 (7)	0.26565 (19)	0.0237 (8)	
H3A	0.4265	0.6324	0.2226	0.028*	
C4	0.43547 (15)	0.6575 (7)	0.3514 (2)	0.0269 (8)	
C5	0.41094 (16)	0.5505 (7)	0.4151 (2)	0.0285 (9)	
H5A	0.4292	0.6107	0.4733	0.034*	

### Atomic displacement parameters (Å^2^)

**Table:**

	*U*^11^	*U*^22^	*U*^33^	*U*^12^	*U*^13^	*U*^23^
Ag1	0.05584 (14)	0.04198 (16)	0.02679 (13)	0.01450 (12)	0.02051 (11)	0.00050 (10)
Br1	0.03597 (16)	0.0366 (2)	0.03715 (17)	−0.00643 (15)	0.01160 (13)	−0.00657 (15)
S1	0.0333 (3)	0.0199 (4)	0.0160 (3)	0.0027 (3)	0.0060 (3)	−0.0009 (3)
N1	0.0360 (12)	0.0253 (13)	0.0241 (11)	−0.0001 (12)	0.0120 (9)	−0.0010 (11)
O1	0.0639 (13)	0.0433 (10)	0.0244 (9)	−0.0083 (12)	0.0236 (9)	0.0060 (9)
O2	0.0498 (13)	0.0449 (14)	0.0294 (12)	0.0224 (13)	−0.0071 (11)	−0.0057 (12)
O3	0.0608 (13)	0.0185 (11)	0.0306 (10)	−0.0012 (11)	0.0164 (10)	−0.0084 (9)
C1	0.0207 (11)	0.0258 (16)	0.0285 (14)	−0.0027 (12)	0.0075 (11)	0.0065 (12)
C2	0.0308 (12)	0.0156 (13)	0.0149 (11)	0.0024 (12)	0.0091 (10)	0.0012 (11)
C3	0.0241 (11)	0.0291 (17)	0.0216 (12)	0.0056 (12)	0.0126 (10)	0.0014 (12)
C4	0.0160 (11)	0.0322 (17)	0.0297 (15)	0.0008 (14)	0.0044 (11)	0.0033 (14)
C5	0.0294 (13)	0.0385 (19)	0.0159 (13)	0.0021 (15)	0.0056 (11)	0.0012 (13)

### Geometric parameters (Å, °)

**Table:**

Ag1—N1^i^	2.270 (3)	N1—C5	1.344 (4)
Ag1—O3^ii^	2.352 (3)	N1—Ag1^iv^	2.270 (3)
Ag1—O2^iii^	2.488 (3)	O2—Ag1^iii^	2.488 (3)
Ag1—O1	2.552 (3)	O3—Ag1^v^	2.352 (3)
Ag1—Ag1^iii^	3.0159 (8)	C1—C2	1.411 (5)
Br1—C4	1.894 (3)	C1—H1A	0.9300
S1—O2	1.437 (3)	C2—C3	1.370 (4)
S1—O3	1.462 (3)	C3—C4	1.374 (4)
S1—O1	1.463 (3)	C3—H3A	0.9300
S1—C2	1.785 (3)	C4—C5	1.388 (5)
N1—C1	1.297 (4)	C5—H5A	0.9300
			
N1^i^—Ag1—O3^ii^	165.92 (9)	S1—O1—Ag1	113.87 (15)
N1^i^—Ag1—O2^iii^	88.66 (10)	S1—O2—Ag1^iii^	143.5 (2)
O3^ii^—Ag1—O2^iii^	98.22 (9)	S1—O3—Ag1^v^	110.48 (15)
N1^i^—Ag1—O1	88.93 (10)	N1—C1—C2	122.2 (3)
O3^ii^—Ag1—O1	88.52 (8)	N1—C1—H1A	118.9
O2^iii^—Ag1—O1	160.60 (9)	C2—C1—H1A	118.9
N1^i^—Ag1—Ag1^iii^	119.93 (7)	C3—C2—C1	118.6 (3)
O3^ii^—Ag1—Ag1^iii^	74.01 (6)	C3—C2—S1	120.3 (2)
O2^iii^—Ag1—Ag1^iii^	72.45 (7)	C1—C2—S1	120.9 (2)
O1—Ag1—Ag1^iii^	92.22 (6)	C2—C3—C4	118.6 (3)
O2—S1—O3	113.53 (16)	C2—C3—H3A	120.7
O2—S1—O1	113.59 (17)	C4—C3—H3A	120.7
O3—S1—O1	112.07 (17)	C3—C4—C5	119.8 (3)
O2—S1—C2	105.49 (15)	C3—C4—Br1	119.8 (3)
O3—S1—C2	106.43 (14)	C5—C4—Br1	120.4 (2)
O1—S1—C2	104.83 (15)	N1—C5—C4	120.8 (3)
C1—N1—C5	120.0 (3)	N1—C5—H5A	119.6
C1—N1—Ag1^iv^	123.1 (2)	C4—C5—H5A	119.6
C5—N1—Ag1^iv^	116.9 (2)		

Symmetry codes: (i) *x*, −*y*+1, *z*−1/2; (ii) *x*, *y*+1, *z*; (iii) −*x*+1/2, −*y*+3/2, −*z*; (iv) *x*, −*y*+1, *z*+1/2; (v) *x*, *y*−1, *z*.

### Hydrogen-bond geometry (Å, °)

**Table:**

*D*—H···*A*	*D*—H	H···*A*	*D*···*A*	*D*—H···*A*
C3—H3A···Br1^vi^	0.93	2.92	3.832 (3)	168

Symmetry codes: (vi) −*x*+1, *y*, −*z*+1/2.
